# LncRNA XIST/miR-200c regulates the stemness properties and tumourigenicity of human bladder cancer stem cell-like cells

**DOI:** 10.1186/s12935-018-0540-0

**Published:** 2018-03-20

**Authors:** Ran Xu, Xuan Zhu, Fangzhi Chen, Changkun Huang, Kai Ai, Hongtao Wu, Lei Zhang, Xiaokun Zhao

**Affiliations:** Department of Urology, The Second Xiangya Hospital, Central South University, Middle Renmin Road No. 139, Changsha, 410011 Hunan China

**Keywords:** LncRNA, XIST, MiR-200c, Bladder cancer, Stem cell-like cells, EMT

## Abstract

**Background:**

The abnormal expression of non-coding RNAs (ncRNAs), such as microRNAs and long ncRNAs, often contribute to the development of cancers. miR-200c functions as a tumour suppressor that impacts the growth of bladder cancer cells and the epithelial-to-mesenchymal transition (EMT). LncRNA X inactive specific transcript (XIST) is highly expressed in tumour tissues, promotes cancer progression and might act as an miRNA molecular sponge. This study aimed to examine the relationship between lncRNA XIST and miR-200c and to assess their functions in the regulation of the stemness properties and tumourigenicity of human bladder cancer stem cell (BCSC)-like cells.

**Methods:**

Biological effects including cell clone formation, sphere formation, self-renewal properties and mouse tumourigenesis were examined in BCSC-like cells with miR-200c overexpression or XIST knockdown. Real-time PCR and western blotting were used to detect the expression changing of related factors in BCSC-like cells gene models. Dual luciferase reporter assay was used to examine the changes of XIST and miR-200c expression levels.

**Results:**

The results indicated that miR-200c overexpression and XIST knockdown could inhibit cell clone formation, self-renewal ability and EMT in BCSC-like cells. miR-200c knockdown could restore the tumour growth inhibition caused by XIST knockdown.

**Conclusion:**

LncRNA XIST may act as an inhibitor of miR-200c to regulate the stemness properties and tumourigenicity of bladder cancer cells, and our findings might reveal a potential strategy of targeting XIST for bladder cancer therapy.

**Electronic supplementary material:**

The online version of this article (10.1186/s12935-018-0540-0) contains supplementary material, which is available to authorized users.

## Background

Bladder cancer is one the most common malignancies of the urinary tract and has been responsible for an estimated 165,000 deaths worldwide in 2012 [1]. The incidence of bladder cancer has gradually increased in recent years. There is a modestly effective clinical prognosis despite systemic therapy for bladder cancer that is characterised by progression, metastasis, recurrence and drug resistance [2, 3]. Although a great deal of advanced research has contributed to the understanding of bladder cancer, molecular mechanisms involved in the pathogenesis and progression of this disease remain poorly understood [4]. Therefore, to develop a more effective therapeutic approach for this disease, further exploring the mechanisms involved in the proliferation, metastasis and invasion of bladder cancer is necessary.

MicroRNAs (miRNAs) are one of the small non-coding RNAs (ncRNAs) (approximately 18–22 nucleotides) that negatively regulate the target gene expression by binding to the 3′ untranslated regions of the protein-coding transcripts. These have been increasingly demonstrated to play important roles in tumourigenesis, tumour migration and progression [5, 6]. Numerous studies have demonstrated that miR-200c functions as a tumour suppressor that impacts the cancer cell growth and survival [7, 8]. In different human cancers, such as ovarian [9], breast [10] and prostate [11] cancers, miR-200c expression is obviously reduced. miR-200c expression has also been demonstrated to participate in the regulation of the epithelial-to-mesenchymal transition (EMT), which is a biological process responsible for tumour progression, invasion and migration in bladder cancer cells [12].

Mounting evidence has suggested that the miRNA activity is influenced by long non-coding RNAs (lncRNAs) [13]. LncRNAs are a class of transcripts that are longer than 200 nucleotides and have a limited ability for protein coding. As a potential mechanism, lncRNAs competitively bind to targeted miRNAs by acting as miRNA molecular sponges [14]. This lncRNA–miRNA cross talk plays a critical role in various processes, including proliferation, cell cycle arrest and apoptosis. An abnormal expression of lncRNAs or miRNAs often contributes to the progression, invasion and unrestricted growth of cancer cells.

In this study, we predicted lncRNAs with complementary base pairing with miR-200c using an online software program (http://www.mircode.org/index.php). From the results, we focused on the lncRNA X inactive specific transcript (XIST) that is located on chromosome Xq13.2 and is required for the transcriptional silencing of one of the pair of the X chromosomes of mammalian females during early development. XIST has been identified to be involved in differentiation and proliferation [15]. LncRNA XIST was highly expressed in gliomas [16, 17], and its overexpression is associated with the growth, invasion, metastasis and development of ovarian cancer [18].

The high expression of LncRNA XIST in tumour tissues promotes cancer progression, whereas that of miR-200c inhibits cancer progression. Several studies have reported that lncRNA XIST expression is negatively associated with miR-200c expression in tumour tissues [16, 17]. Therefore, we speculate a competitive relationship between XIST and miR-200c in the regulation of tumour cell occurrence, proliferation and invasion. The mechanism and function of XIST and miR-200c in the pathogenesis of bladder cancer remain largely unknown. To address these gaps, we utilised bladder cancer stem cell (BCSC)-like cells from cell lines 5637 and T24 and isolated the cancer stem cells. The objectives were to detect biological effects of BCSCs with miR-200c overexpression or with XIST knockdown. Our present study suggests that lncRNA XIST knockdown inhibits the stemness properties and tumourigenicity by sponing miR-200c in BCSC-like cells and reveals a potential strategy of targeting XIST for bladder cancer therapy.

## Methods

### Cell culture and transfection

Human bladder cancer cell lines 5637 and T24 were purchased from the Cell Centre of the Xiangya School of Medicine, Central South University (Hunan, China). The cells were cultured in DMEM medium (Gibco, California, USA), supplemented with 10% fetal bovine serum (Gibco). All cell lines were maintained in an incubator with a humidified atmosphere of 5% CO_2_ at 37 °C.

The specific pRNAT-U6.1/Neo vector encoding a short hairpin RNA targeting the lncRNA XIST was named anti-XIST (sequences: 5′-GCU GCU AGU UUC CCA AUG AUA-3′), meaningless small fragments of equal length were used to construct negative control plasmid named anti-control (sequences: 5′-UUC UCC GAA CGU GUC ACG UTT-3′). miR-200c mimics (Catalog#: HmiR-SN0301), miR-200c inhibitor (Catalog#: HmiR-AN0301), mimics scramble (Catalog#: CmiR-SN0001) and inhibitor scramble (Catalog#: CmiR-AN0001) purchased from GeneCopoeia (Maryland, USA) were used to construct the overexpression and knockdown models, as well as negative controls. The plasmid, mimics, inhibitor and their negative controls were transfected into 5637 and T24 cells, respectively, using the Lipofectamine^2000^ transfection reagent (Invitrogen, California, USA) according to the manufacturer’s protocol.

### Sphere formation, cell clone formation and self-renewal assay

The BCSC-like cells of 5637 and T24 were sorted by spherocyst medium from the cell lines. Briefly, single cell suspensions of 5637 and T24 were planted in ultra-low adhesion, six-well plates and grown in a stem cell growth medium containing 1× DMEM/F12 (Gibco), 1× B27 (Invitrogen), 20 ng/mL of epidermal growth factor (Gibco), 20 ng/mL of fibroblast growth factor (Gibco), 0.4% bovine serum albumin (Gibco) and 4 μg/mL of insulin. Western Blotting was used to identify the BCSC-like cells, testing the protein expression levels of specific stem cell markers. The growth of spheres was observed under a phase contrast microscope (Olympus, Tokyo, Japan).

The target cancer stem cells, which were separated using magnetic bead assays, were seeded in ultra-low adhesion, six-well plates at a density of 2 × 10^4^ cells/well. After 6–8 days in sphere formation culture, the anti-XIST plasmid or the miR-200c overexpression plasmid was transfected in sphere forming cells using the transfection reagent. After 2 or 3 weeks, the number of spheres (> 10 cells) was quantified under an inverted microscope (Olympus). The cloning forming efficiency was calculated as follows: the number of spheres/the quantity of planted cells) × 100%.

Cell self-renewal assays were performed using ultra-low adhesion, 96-well plates containing 200 μL stem cell growth medium at a density of 1 cell/well after transfection with the anti-XIST plasmid or the miR-200c overexpression plasmid. The number of cells was counted after 6–10 days in culture. The efficiency of self-renewal was calculated as follows:

$${\text{the number of cells}}/{\text{the number of seeded cells}}) \times 100\% .$$

### Quantitative real-time PCR analysis (qPCR)

Total RNA was extracted from cell lines using the Trizol reagent (Invitrogen), and cDNA was synthesised from total RNA using the SuperScript III (Invitrogen) according to the manufacturer’s instructions. qPCR was performed using the Real-Time Quantitative PCR SYBR Green kit (Takara, Tokyo, Japan). Primer sequences used were *β*-actin, forward 5′-AGG GGC CGG ACT CGT CAT ACT-3′ and reverse 5′-GGC GGC ACC ACC ATG TAC CCT-3′; XIST, forward 5′-GCT CTT CAT TGT TCC TAT CTG CC-3′ and reverse 5′-TGT GTA AGT AAG TCG ATA GGA GT-3′. miR-200a (Catalog#: HmiRQP0297), miR-200b (Catalog#: HmiRQP0299), miR-200c (Catalog#: HmiRQP0301) and U6 (Catalog#: HmiRQP9001) were purchased from GeneCopoeia. The relative expression of each gene was calculated using the 2^−ΔΔCT^ method relative to the expression levels of β-actin or U6.

### Western blotting

Cells were harvested and lysed. After the supernatants were collected, equal amounts of total protein (30 μg) were separated using 12% SDS-PAGE and transferred to a polyvinylidene fluoride membrane by electroblotting. The membranes were blocked in 5% non-fat milk for 1 h and incubated at 4 °C overnight with primary antibodies: mouse monoclonal anti-CD133 (1:500, ABZOOM, Texas, USA), mouse monoclonal anti-KLF4 (1:1000, ABZOOM), rabbit polyclonal anti-OCT-4 (1:1000, Proteintech, Chicago, USA), mouse monoclonal anti-CD44 (1:1000, ABZOOM), mouse monoclonal anti-ABCG2 (1:1000, ABZOOM), mouse monoclonal anti-E-cadherin (1:50, Abcam, Cambridge, UK), rabbit monoclonal anti-vimentin (1:2000, Abcam), mouse monoclonal anti-ZEB1 (1:1000, ABZOOM), rabbit polyclonal anti-ZEB2 (1:500, Abcam) and mouse monoclonal anti-β-actin (1:2000, Abcam). Bound signals were visualised after incubation with HRP-conjugated goat anti-mouse (1:18,000, Auragene, Changsha, China) or goat anti-rabbit (1:1500, Auragene) secondary antibody and exposure to X-ray film. Scanned images were analysed using ImageJ software.

### Dual luciferase reporter assay

The 5637 and T24 cell lines were seeded in a six-well dish at a density of 2 × 10^5^ cells. The psiCHECKTM-2 vector with the cloned miR-200c binding site of XIST, named XIST-wild type (WT), was co-transfected with miR-200c mimics or mimics NC using the Lipofectamine^2000^ transfection reagent. The mutation-carrying psiCHECKTM-2 vector with a different sequence of the miR-200c binding site, named as the XIST-mutation (Mut), as the control group, was constructed using the QuickChange Site-Directed Mutagenesis Kit (Stratagene, California, USA). The luciferase activity was measured 48 h after transfection using the Dual Luciferase Reporter Assay Kit (Promega, Wisconsin, USA).

### Mouse tumourigenesis assay

All animal assays were conducted adhering to the rules of the Animal Ethics and Welfare Committee of the Second Xiangyang Hospital of Central South University (Hunan, China). The T24 sphere forming cells (1 × 10^7^) transfected with anti-XIST plasmid and miR-200c inhibitor were subcutaneously injected into the left and right flanks of 6-week-old NOD/SCID mice that were purchased from the Laboratory Animal Centre of Xiangya Hospital of Central South University. After 6 weeks of tumourigenesis in vivo, the tumour tissue was harvested and weighed and immunohistochemistry (IHC) was performed by hematoxylin & eosin, Ki67 and E-cadherin staining. Tumor volume was calculated by using the formula ${\text{V}}\left( {mm3} \right) = 0.2618 \times {\text{a}} \times {\text{b}} \times ({\text{a}} + {\text{b}})$ (a, maximum length to diameter; b, maximum transverse diameter).

### Immunohistochemical (IHC) staining

All tissue samples were divided into three slice. Slides were deparaffinized in dimethylbenzene, hydrated in an alcohol gradient, endogenous peroxidase was inactivated, in 3% hydrogen peroxide, and then retrieved in citric acid buffer (pH6.0) in a pressure cooker for 3 min. After cooling, the slices were blocked with normal goat serum, and incubated with primary mouse monoclonal anti-Ki67 (1:100, Abcam) or mouse monoclonal anti-E-cadherin (1:50, Abcam) antibody overnight at 4 °C. The slides were then incubated with goat anti-mouse secondary antibody (Ready to use, Auragene, Changsha, China) for 2 h at 37 °C. The sections were then stained with a DAB Detection Kit (Solarbio, Beijing, China) and were counterstained with hematoxylin. Finally, the dyed sections were observed under a microscope.

### Statistical analysis

Statistical analysis was performed using SPSS 16.0 and GraphPad Prism 5.0 software. Data are represented as mean ± SD. Significant differences in the continuous data between groups were compared using one-way ANOVA and two-tailed Student’s *t* test. *P* < 0.05 was considered statistically significant. *P < 0.05, **P < 0.01.

## Results

### Tumour sphere formation of human bladder cancer 5637 and T24 cells

**Fig. 1:**
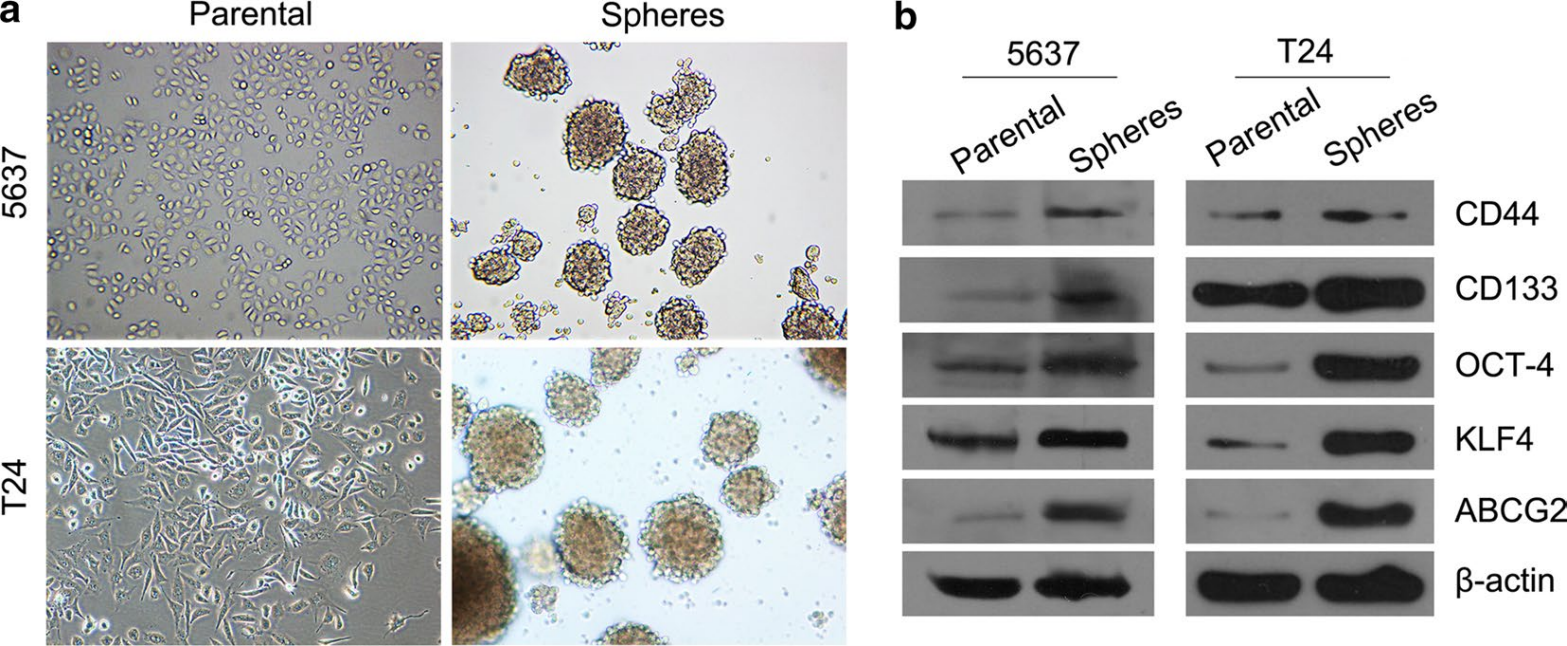
Sphere formation of human bladder cancer stem cell-like side population cells. **a** The tumour sphere formation of human bladder cancer 5637 and T24 cells compared with that of the parental cells (magnification, 100×). **b** Western blotting of CD133, CD44, KLF4, OCD-4 and ABCG2 protein expression in parental and sphere 5637 and T24 cells

To explore the effect of miR-200c and lncRNA XIST in the human bladder cancer 5637 and T24 cell lines, the maintenance of the stemness properties of BCSC-like cells was sorted by spherocyst medium and then performing sphere formation assays (Fig. 1a).

To investigate whether the BCSC-like cells of 5637 and T24 possess the stemness properties, Western blotting was performed to compare the expression levels of BCSC markers such as CD133 and CD44 between the parental and sphere cells. The protein expression of CD133, CD44, KLF4, OCT-4 and ABCG2 was higher in the BCSC sphere cells compared to the parental cells (Fig. 1b).

### miR-200c has a low expression and XIST has a high expression in the sphere forming cells compared to the parental cells

**Fig. 2:**
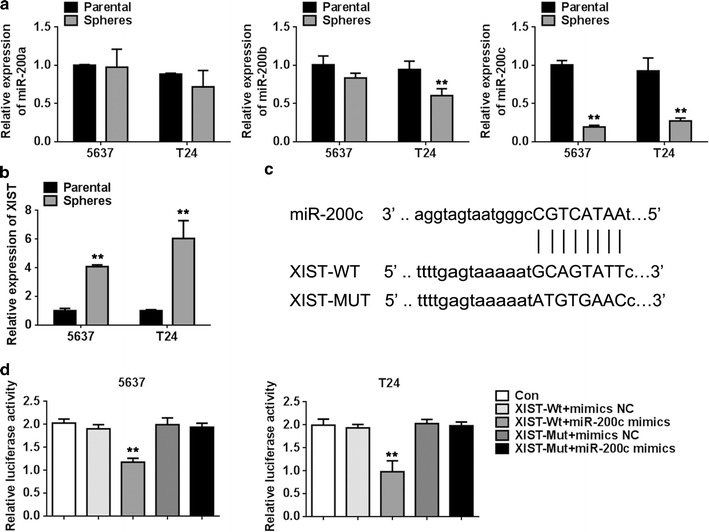
Targeting relationship between miR-200c and XIST. **a** The relative mRNA expression level of miR-200 was detected using qPCR in sphere and parental cells. **b** The relative mRNA expression level of XIST was detected using qPCR in bladder cancer stem cell-like side population cells and parental cells. **c** The targeted binding sites of miR-200c and XIST. **d** The dual luciferase reporter assays showed that the relative luciferase activity of 5637 and T24 cells co-transfected with XIST-Wt and miR-200c was dramatically decreased compared with that of the control group. Data are presented as mean ± SD. **P < 0.01 vs. parental or control group

qPCR revealed decreased mRNA expression levels of miR-200a, miR-200b, miR-200c (Fig. 2a) in the sphere forming cells compared to the parental cells in 5637 and T24 cell lines. Only the relative expression of miR-200c was significantly decreased in the BCSC sphere cells compared to the parental cells in the 5637 and T24 cell lines. These results suggested that miR-200c had the lowest expression in human BCSC-like cells.

In contrast, several studies have reported the high expression of lncRNA XIST in several tumour tissues such as glioma [16, 17] and ovarian cancer [18]. Indeed, our study indicated that the mRNA expression of XIST was significantly higher (Fig. 2b) in the BCSC sphere cells compared to the parental cells by qPCR. Furthermore, our software analysis revealed a binding site between miR-200c and XIST (Fig. 2c). These evidences may suggest a relationship between miR-200c and XIST influencing the biological functions of bladder cancer cells.

To identify whether miR-200c has a function in targeting XIST, dual luciferase reporter assays were performed. We cloned the predicted miR-200c binding site of XIST, named as XIST-Wt, and a mutated targeting site of XIST, named as XIST-Mut vector. The results showed a dramatically decreased relative luciferase activity in 5637 and T24 parental cells co-transfected with XIST-Wt and miR-200c and no significant changes in the group co-transfected with XIST-Wt and miR-NC and in the group co-transfected with XIST-Mut and miR-200c or miR-NC (Fig. 2d). These results suggest that XIST regulates BCSC-like cells by functioning as a molecular sponge of miR-200c.

### miR-200c overexpression inhibited the cell clone formation and self-renewal properties of BCSC-like cells

**Fig. 3:**
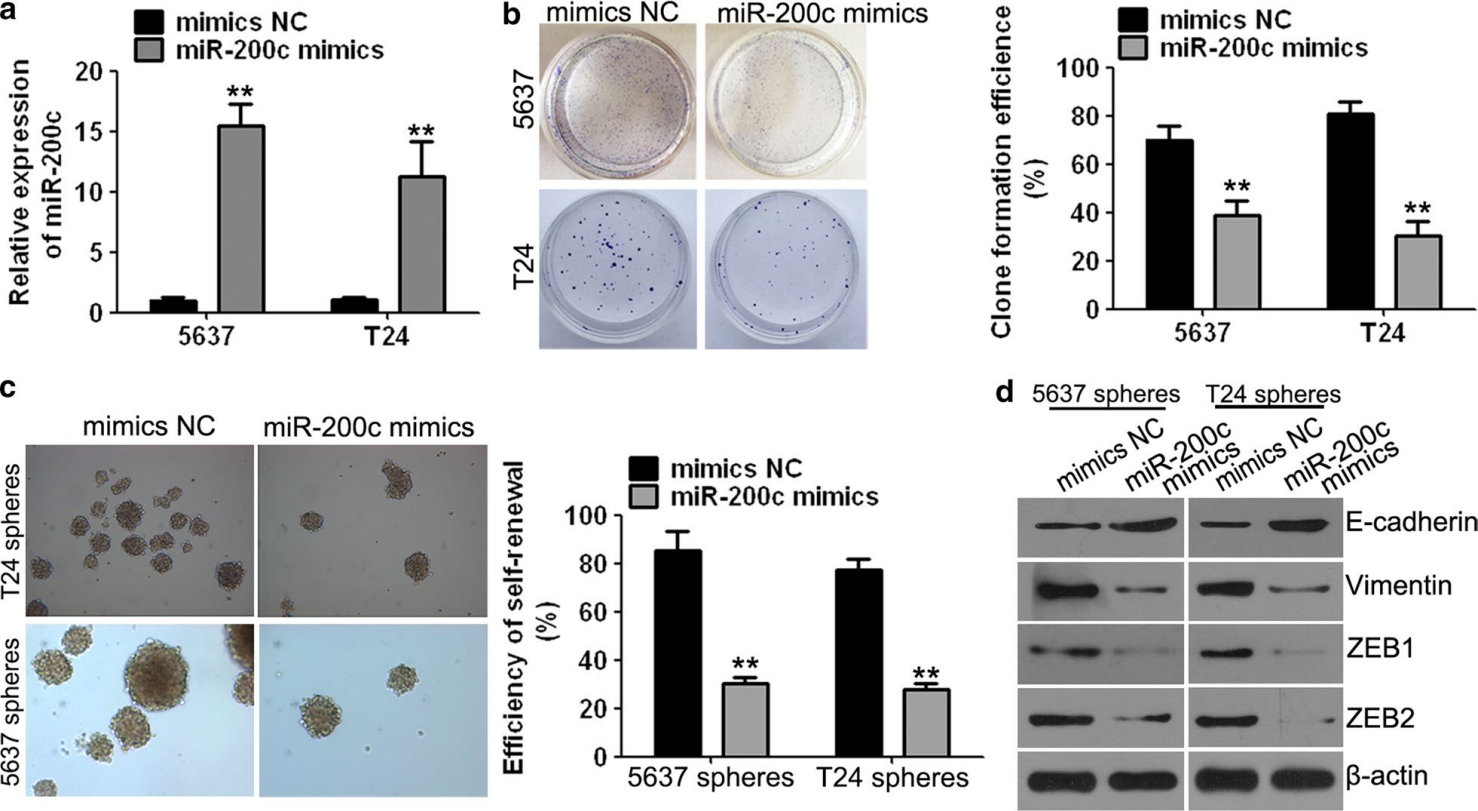
miR-200c mimics inhibited clone formation and self-renewal capacities in cancer stem cell-like side population cells. **a** qPCR assays were performed to assess the available 5637 and T24 bladder cancer stem cell-like side population cells transfected with miR-200c mimics and negative control (NC). **b** Cell clone formation assays demonstrated that the clone formation ability of 5637 and T24 cells was significantly decreased in the miR-200c mimics group compared to the NC group. **c** The self-renewal capacity of bladder cancer stem cell-like side population cells (magnification, 100×). **d** miR-200c mimics affected the expression of EMT-associated factors such as E-cadherin, vimentin and ZEB1 and ZEB2 proteins. Data are presented as mean ± SD. **P < 0.01 vs. mimics NC group

To explore the effect of miR-200c on the proliferation and metastasis in the BCSC-like cells, we transfected the first passage of BCSC-like 5637 and T24 cells with the miR-200c mimics (the miR-200c mimics group) or negative control (the mimics NC group). qPCR assays were used to confirm the available BCSC-like cell models transfected with miR-200c mimics. The relative mRNA level of miR-200c was significantly higher in the miR-200c mimics group compared to the mimics NC group (Fig. 3a). The miR-200c overexpression model was successfully constructed.

Clone formation efficiencies of 5637 and T24 cells were dramatically reduced compared with those of the mimics NC group when cells were treated with miR-200c mimics (Fig. 3b). These findings suggest that miR-200c inhibited the growth in human bladder cancer cells.

The self-renewal capacity, which is a characteristic of cancer stem cells [19], was detected by the ability of human bladder cancer 5637 and T24 cells to form tumour spheres. The assay showed that the self-renewal capacity of BCSC-like 5637 and T24 cells was significantly inhibited in the miR-200c mimics group compared to the mimics NC group (Fig. 3c). These results suggested that miR-200c inhibits the self-renewal ability of BCSC-like cells.

In addition, we detected that the expression of EMT-associated factors such as E-cadherin was significantly increased and that of vimentin, ZEB1 and ZEB2 was significantly decreased in the miR-200c mimics group compared to the mimics NC group (Fig. 3d).

### XIST knockdown inhibited the cell clone formation and self-renewal properties of BCSC-like cells

**Fig. 4:**
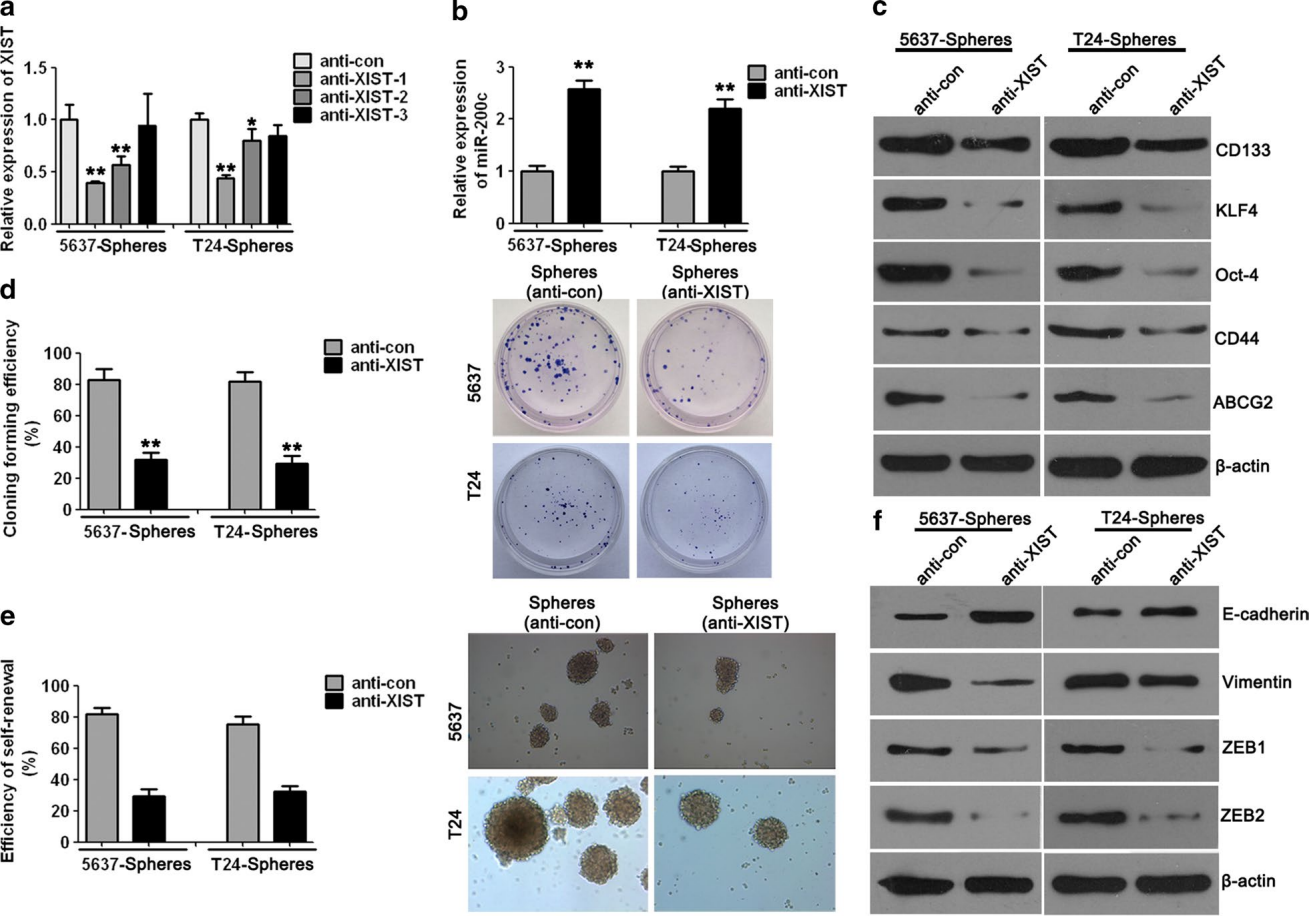
XIST knockdown inhibited cell clone formation and self-renewal in cancer stem cell-like side population cells. **a** qPCR analysis was performed to detect the effective interference plasmids targeting XIST. **b** miR-200c was significantly increased in the XIST knockdown group compared to the control group. **c** The protein expression of BCSC markers was decreased after XIST interference tested by Western blotting. **d** The efficiency of clone formation was significantly decreased in the anti-XIST group compared to the anti-con group. **e** The self-renewal capacity of bladder cancer stem cell-like side population cells was dramatically decreased when cells were treated with anti-XIST than when treated with anti-con (magnification, 100×). **f** The protein expression levels of EMT-associated factors in bladder cancer stem cell-like side population cells detected by Western blotting. Data are presented as mean ± SD. *P < 0.05, **P < 0.01 vs. anti-con group

To explore the function of lncRNA XIST in the BCSC-like cells, we designed and constructed three anti-XIST plasmids, named as anti-XIST-1, anti-XIST-2 and anti-XIST-3, targeting different sequence regions of XIST. Their negative control (NC) plasmids were named as anti-con. qPCR analysis was performed to detect the effective interference of plasmid encoded targets for XIST (Fig. 4a). The relative expression of XIST was significantly decreased in the group treated with anti-XIST-1, and anti-XIST-1 was chosen for further analysis.

To further ascertain a functional relationship between miR-200c and XIST, the relative expression of miR-200c was detected using qPCR and shown to be significantly increased after XIST knockdown compared with that in the control group both in 5637 and T24 cells (Fig. 4b).

We further detected the protein expression of BCSC markers, including CD133, CD44, KLF4, OCT-4 and ABCG2, between the XIST interference group and control group by Western blotting. The analysis showed that the protein expression of CD133, CD44, KLF4, OCT-4 and ABCG2 was decreased after XIST knockdown compared with that in the control group both in 5637 and T24 BCSC-like cells (Fig. 4c).

We transfected the first passage of BCSC-like cells with the anti-XIST plasmid or anti-con to explore the effect of XIST on proliferation and metastasis in the 5637 and T24 BCSC-like cells. The clone formation efficiency of 5637 and T24 was dramatically decreased when the cells were treated with anti-XIST plasmid compared to cells that were treated with anti-con (Fig. 4d).

To explore whether XIST regulates the self-renewal capacity of 5637 and T24 BCSC-like cells, a further self-renewal assay was performed to detect the self-renewal capacity of BCSC-like cells. The results showed that the reduced expression levels of XIST inhibit the self-renewal capacity of the 5637 and T24 BCSC-like cells, and the efficiency of self-renewal was significantly lower when the cells were treated with the anti-XIST plasmid in contrast to those treated with anti-con (Fig. 4e). These results suggested that XIST functions in regulating the self-renewal capacity of the 5637 and T24 BCSC-like cells.

Furthermore, we identified the protein expression levels of EMT-associated factors using Western blotting in the 5637 and T24 BCSC-like cells in which XIST was inhibited. Compared with the control group, the expression of E-cadherin was increased and that of vimentin, ZEB1 and ZEB2 were decreased in the anti-XIST group (Fig. 4f). These results revealed that XIST knockdown inhibits EMT in BCSC-like cells.

### XIST and miR-200c are associated with tumour growth and proliferation in vivo

**Fig. 5:**
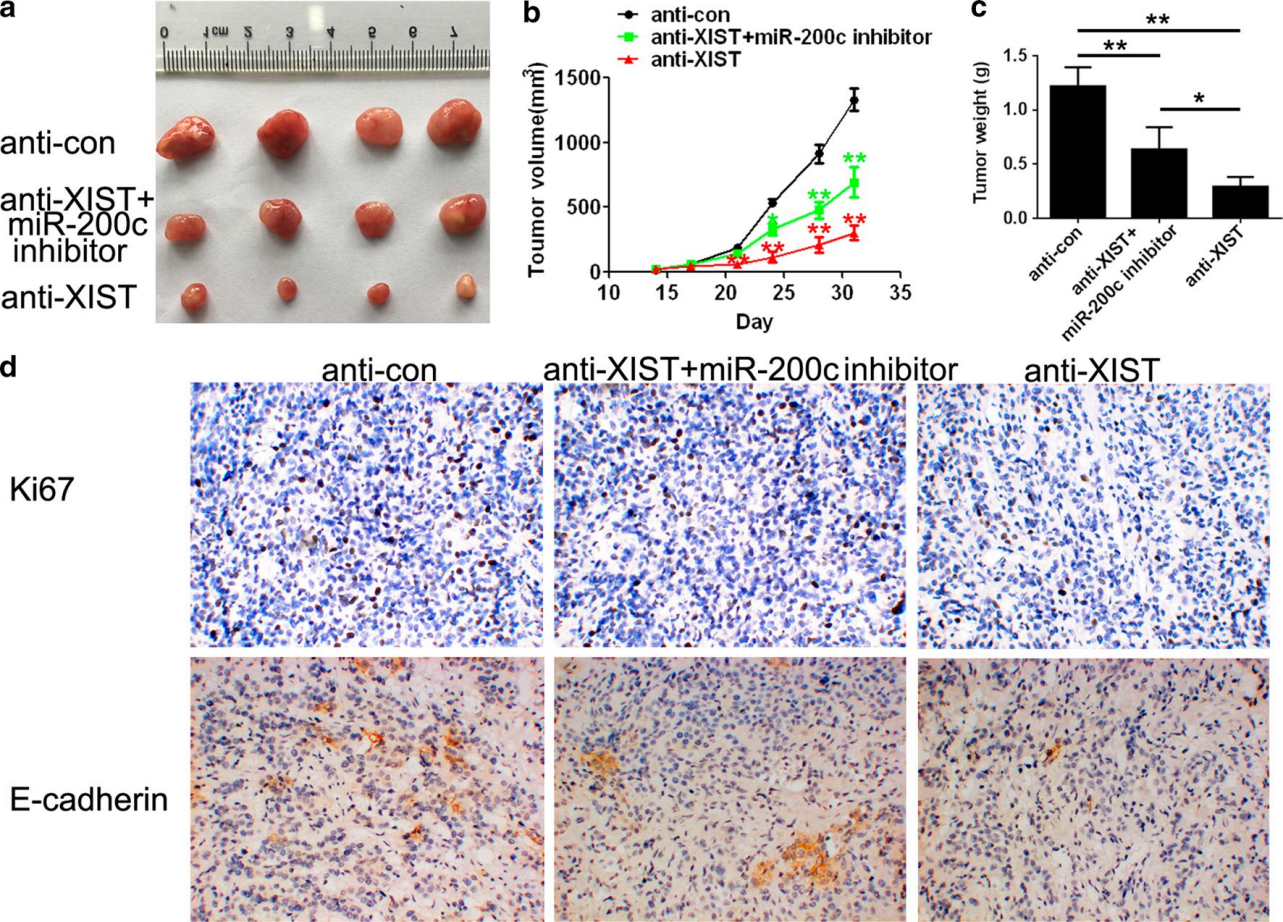
XIST and miR-200c regulated the tumour growth and proliferation in vivo. **a** The tumours harvested from mice. **b** The tumour volume of xenografts. **c** The weight of tumours. **d** The expression of Ki67 and E-cadherin detected by immunohistochemistry, and the expression was lower in the treated group compared to the anti-con group (magnification, 200×). Data are presented as mean ± SD. *P < 0.05, **P < 0.01 vs. anti-con group

To investigate whether XIST and miR-200c are associated with tumour growth, mouse tumourigenesis assays were performed. Compared with the control group, xenografts in the anti-XIST group were much smaller and lighter, and co-transfection with miR-200c inhibitor could partially revert the inhibition of tumour growth (Fig. 5a–c). The modeling results of miR-200c inhibitor and the expression of miR-200c after tumorigenesis in nude mice are described in Additional file 1. To explore the effects of XIST and miR-200c expression on proliferation and EMT, IHC assays were performed to detect the expression of Ki67 and E-cadherin. The amount of Ki67-positive stained cells was significantly higher in the anti-XIST group compared to the control group (Fig. 5d). XIST knockdown dramatically increased the expression of EMT-associated factor E-cadherin compared with that in the control group (Fig. 5e), whereas co-transfection with miR-200c inhibitor partially restored the expression levels of this protein.

## Discussion

Approximately 70% of the human genome is transcribed, whereas only 2% encodes protein [20]. Based on their sizes, ncRNAs can be grouped into lncRNAs (> 200 bp) and small ncRNAs (≤ 200 bp), such as miRNAs. ncRNAs play crucial roles in various biological processes such as differentiation, apoptosis and proliferation. miR-200c and lncRNA XIST have been reported to affect the biological functions of multiple cancer cells including gastric cancer and breast cancer cells, respectively [21, 22]. The effect of miR-200c and lncRNA XIST in bladder cancer and a potential relationship between miR-200c and XIST remain largely unknown. Therefore, this study aimed to explore the function and regulatory mechanism of XIST and miR-200c in the maintenance of the stemness properties and tumourigenicity of human bladder cancer cells.

A great deal of evidence has confirmed the existence of cancer stem cells, which possess the capacities for tumour cell initiation, proliferation, differentiation and self-renewal [2, 23]. The commonly reported specific surface markers, such as CD44, CD133, OCT-4, Bmi-1, ALDH1, ABCG2 and KLF4, with high expressions, are usually used to isolate cancer stem cells from cell lines [24, 25]. The routinely employed method of cancer stem cell identification is the sorting of BCSC-like cells by spherocyst medium followed by the isolation of the cancer stem cells according to their specific markers, such as CD133 and CD44. The self-renewal and clone formation assays usually follow to explore the stemness properties of the cells.

The cancer stem cells were isolated from cell lines 5637 and T24 in the present study to better understand the biological function and regulatory mechanisms of lncRNA XIST and miR-200c in human bladder cancer cells. The expression of CD44, CD133, OCT-4, KLF4 and ABCG2 was studied to verify the BCSC-like 5637 and T24 cells using Western blotting. The results revealed the expression of CD44, CD133, OCT-4, KLF4 and ABCG2 in the sphere forming cells and verified that cancer stem cells displaying the stemness properties were successfully isolated. Furthermore, we used the sphere forming 5637 and T24 cells as BCSC models to investigate their self-renewal and clone formation capacities under miR-200c overexpression or XIST knockdown condition. Our results demonstrated that the self-renewal and clone formation capacities were significantly decreased with miR-200c overexpression and XIST knockdown in the BCSC-like 5637 and T24 cells. These data revealed that miR-200c functions in inhibiting the stemness properties and that lncRNA XIST possesses the reverse function in bladder cancer cells.

Epithelial-to-mesenchymal transition is an important cellular mechanism in several biological processes, such as embryonic development, chronic inflammation, tissue repair and tumour metastasis, that involves the loss of intercellular adhesion and acquisition of an invasive and migratory mesenchymal phenotype [26, 27]. During EMT, cells lose epithelial characteristics such as the down-regulation of E-cadherin, which is one of the most commonly reported epithelial cell markers, and gain a mesenchymal phenotype with the high expression of mesenchymal proteins, including vimentin, ZEB1 and ZEB2 [28, 29]. EMT process that involves the loss of intercellular adhesion has been extensively associated with metastatic progression in various cancers. Therefore, the EMT process that is associated with the expression of E-cadherin, vimentin, ZEB1 and ZEB2 is generally used to indicate the ability of cancer cell metastasis and invasion.

In the present study, which aimed to detect the metastasis potential of BCSC 5637 and T24 under miR-200c overexpression or XIST knockdown condition, we detected the protein expression of E-cadherin, vimentin, ZEB1 and ZEB2, which are known as EMT-specific markers, by Western blotting. The results suggested that the expression of E-cadherin was increased and that of vimentin, ZEB1 and ZEB2 were decreased in the BCSC-like 5637 and T24 cells under miR-200c overexpression and XIST knockdown compared with cells in their respective control groups. These data indicate that EMT processes that reflect the ability of metastasis and invasion are inhibited by miR-200c overexpression and XIST knockdown.

Increasing evidence has supported that lncRNAs function as effective therapeutic targets for the treatment of cancers, such as prostate, breast and gastric cancers [7, 30]. The abnormal expression of lncRNAs may contribute to cancer occurrence, progression, metastasis and invasion. One of the most important mechanisms of lncRNAs was reported to be their function as miRNA sponges and their competition with protein-coding transcripts for microRNA binding [14, 31]. Each lncRNA can compete for the binding of multiple miRNAs, and similarly each miRNA can target multiple lncRNAs.

miR-200c was found to have a low expression in BCSC-like cells that possess stemness properties. The efficiency of clone formation and self-renewal and the phenomenon of EMT were decreased after miR-200c overexpression. These results suggested that miR-200c inhibits the proliferation, metastasis and migration in human bladder cancer cells. Our data also confirmed that XIST has higher expression in BCSC-like cells compared to their parental cells. XIST knockdown decreased the efficiency of clone formation, self-renewal and EMT, and these results indicated that the low expression levels of XIST inhibit proliferation, metastasis and migration. In addition, the in vivo tumourigenesis assay also demonstrated that XIST knockdown resulted in a dramatic decrease in the size of tumour growth. Together with the results of IHC assays, these data suggest the growth-promoting effect of lncRNA XIST both in vitro and in vivo in human bladder cancer.

LncRNA XIST has been reported to function as a molecular sponge of miR-101 to promote cancer cell progression in gastric cancer [32]. Another study has revealed that XIST promotes gastric cancer proliferation and invasion through sponging miR-497 [30]. In the present study, we found that XIST expression was negatively correlated with miR-200c expression in human BCSC-like cells. Moreover, the results suggested that lncRNA XIST performs the exact opposite function as miR-200c in regulating the proliferation and metastasis of bladder cancer cells. Together with the results of the dual luciferase reporter assay, this demonstrated that XIST directly forms a complementary base pair with miR-200c and acts as a molecular sponge of miR-200c to regulate the biological functions of bladder cancer cells.

## Conclusion

Our results demonstrated that miR-200c can inhibit the stemness properties such as self-renewal, clone formation and EMT in human bladder cancer cells. In contrast, lncRNA XIST has positive effects on the stemness properties and tumourigenicity of human bladder cancer cells. In addition, we assumed that lncRNA XIST performed these functions by acting as a molecular sponge of miR-200c in human bladder cancer cells. Based on the results from our study investigating the underlying mechanism of XIST and miR-200c in regulating bladder cancer cell growth and invasion, XIST might be considered as a potential target for suppressing the proliferation, metastasis and tumourigenicity of human BCSCs.

## Additional file

**Additional file 1.** The relative mRNA expression levels of miR-200c in T24 sphere forming cells (A) and mouse tissues (B). **P < 0.01 vs. control group.

## Abbreviations

ncRNAs: non-coding RNAs
miRNAs: microRNAs
lncRNAs: long non-coding RNAs
EMT: epithelial-to-mesenchymal transition
BCSCs: bladder cancer stem cells
XIST: X inactive specific transcript
qPCR: quantitative real time-PCR analysis
