# Supplementary figures and images for: LncRNA XIST/miR-200c regulates the stemness properties and tumourigenicity of human bladder cancer stem cell-like cells
Source: Cancer Cell Int. 2018 Mar 20;18:41. doi: 10.1186/s12935-018-0540-0 (PMC5859407; doi:10.1186/s12935-018-0540-0)

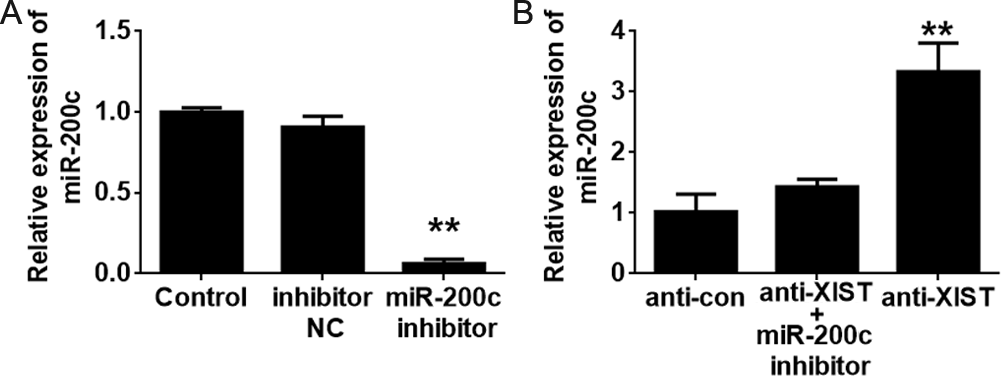

Supplement: Supplementary file 1 — Additional file 1. The relative mRNA expression levels of miR-200c in T24 sphere forming cells (A) and mouse tissues (B). **P < 0.01 vs. control group. [file 12935_2018_540_MOESM1_ESM.tif]
